# Supplementary material for: Tunable metasurfaces via subwavelength phase shifters with uniform amplitude
Source: Sci Rep. 2017 Jan 5;7:40174. doi: 10.1038/srep40174 (PMC5215393; doi:10.1038/srep40174)
Supplement: Supplementary Information [file srep40174-s1.pdf]

Tunable metasurfaces via subwavelength phase shifters with uniform amplitude

Shane Colburn[1], Alan Zhan[2], and Arka Majumdar[1,2]

[1] Department of Electrical Engineering, University of Washington, Seattle.

[2] Department of Physics, University of Washington, Seattle.

Supplementary Information

Asymmetric Fabry-Perot Cavity Analysis

If we model the bottom reflector as a perfect mirror, we can solve for the output E-field as a function of the input E-field, by superposing the initial reflected beam with all of the subsequent beams which pass out of the cavity. If the input wave has amplitude A, the top mirror has external reflectivity r, internal reflectivity r', ingoing transmission amplitude t, outgoing transmission amplitude t', and the phase accumulated in one roundtrip in the cavity is δ, then the initial reflected beam is given by:

$$E_{r1} = Ar, \quad (1)$$

The second outgoing beam passes through the top mirror, picks up a δ phase shift in the cavity, and then passes back out the top mirror, giving:

$$E_{t1} = Att'e^{i\delta}, \quad (2)$$

The third outgoing beam is similar to the second except rather than transmitting through the top after one trip, it takes an additional trip, picking up another δ phase, and scaling by a factor of r':

$$E_{t2} = Att'r'e^{2i\delta}, \quad (3)$$

Extending this to an infinite number of outgoing beams, the total E-field outside the cavity can be expressed as below:

$$E_{total} = Ar + Att'e^{i\delta} \sum_{n=0}^{\infty} \left(r'e^{i\delta}\right)^n, (4)$$

Since $|r'| < 1$, the series converges, and utilizing $tt' - rr' = 1$ and $r = -r'$ by energy conservation, the expression simplifies to:

$$E_{total} = \frac{A\left(r+e^{i\delta}\right)}{1+re^{i\delta}}, (5)$$

In the lossless case, we have $Im\{\delta\} = 0$, and we can find the magnitude and phase of the expression as below:

$$|E_{total}| = A, (6)$$

$$\measuredangle E_{total} = \tan^{-1}\left[\frac{(1-r^2)\sin\delta}{2r+(r^2+1)\cos\delta}\right], (7)$$

In the case with loss, we have $Im\{\delta\} \neq 0$ and we let $\delta = \beta + i\alpha$. The magnitude and phase are then given as below:

$$|E_{total}| = A\sqrt{\frac{e^{-2\alpha}+2re^{-\alpha}cos\beta+r^2}{1+2re^{-\alpha}cos\beta+r^2e^{-2\alpha}}}, (8)$$

$$\measuredangle E_{total} = \tan^{-1}\left[\frac{(1-r^2)\,e^{-\alpha}\sin\beta}{r(1+e^{-2\alpha})+(r^2+1)e^{-\alpha}\cos\beta}\right], (9)$$

We can also determine how rapidly the phase ϕ changes with respect to the cavity phase by taking the derivative and evaluating at $\delta = \pi$, giving:

$$\left.\frac{d\phi}{d\delta}\right|_{\delta=\pi} = \frac{1+r}{1-r}, (10)$$

For the lossy case, we get:

$$\left.\frac{d\phi}{d\beta}\right|_{\beta=\pi} = \frac{(r^2-1)e^{-\alpha}}{r(1+e^{-2\alpha})-(r^2+1)e^{-\alpha}}, (11)$$

By letting $\alpha = 0$, we neglect the loss and recover the lossless equations.

Quality Factor and Loss

The quality factor of a resonator is an important quantity for characterizing resonators and how efficiently they store and dissipate energy. For an asymmetric Fabry-Perot cavity excited by a transient electric field which decays with time, the amplitude within the cavity can be related to the resonator's Q by:

$$E(t) = E_0 e^{-\frac{\omega_0 t}{2Q}}, (12)$$

where t is time, E_0 is the initial electric field, and ω_0 is the resonant angular frequency. In one roundtrip in the cavity, we can express the field amplitude as a product of the top mirror reflectivity r (the bottom mirror has $r = 1$) and the attenuation ρ due to material absorption (i.e. a fraction $1 - \rho$ of the field amplitude is lost per roundtrip) such that:

$$E_{rt} = E_0 r\rho, (13)$$

where E_{rt} denotes the field after one roundtrip. Evaluating (12) at $t = t_{rt}$ and equating to (13), we obtain:

$$Q = \frac{-\omega_0 t_{rt}}{2 \ln r\rho}, (14)$$

Now utilizing the relations $\omega_0 = 2\pi f$ and $t_{rt} = \frac{2Ln}{c}$, where L is the cavity length, n is the refractive index, and c is the speed of light, we can express the Q as below:

$$Q = \frac{-\beta}{2 \ln r\rho}, (15)$$

where, as previously, $\beta = \frac{4\pi nL}{\lambda}$ denotes the phase accumulated in one roundtrip. The value of β is dependent on the structure of the cavity and for an arbitrary value of $\beta = 10$, we plot the Q as a function of the loss fraction $1 - \rho$ for different values of r (Fig. S1). We observe that the Q remains relatively flat for low losses, but decays rapidly as the loss increases.

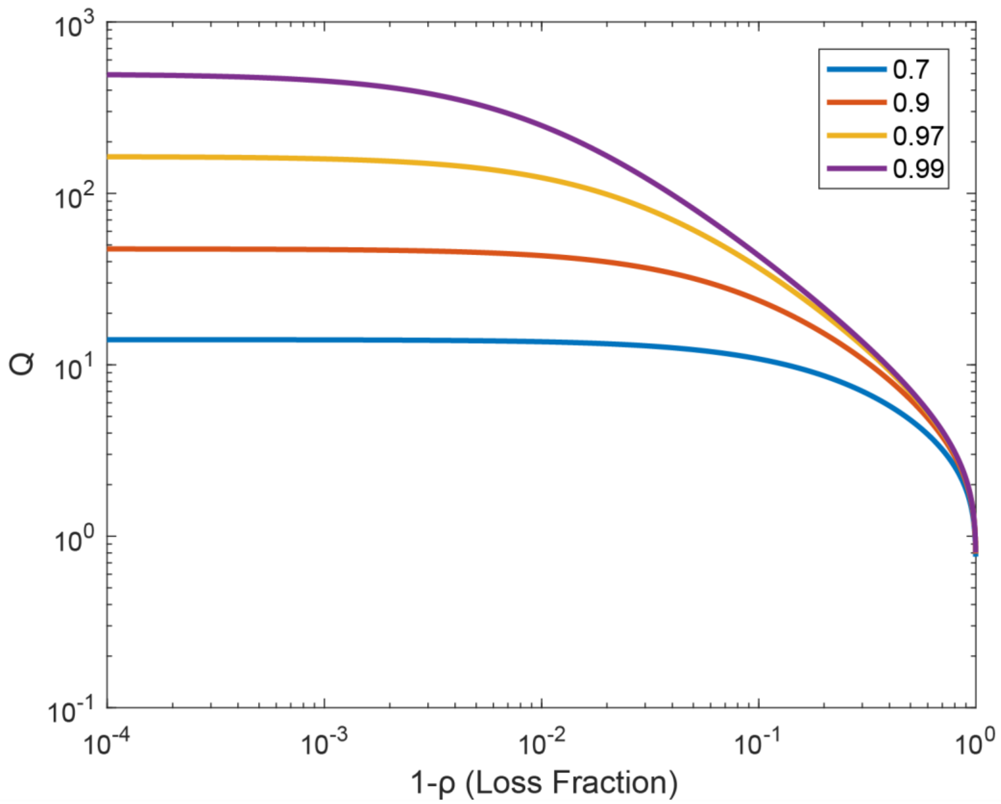

Figure S1: Quality factor of an asymmetric Fabry-Perot cavity with $\beta = 10$ *as a function of the fractional amplitude loss due to material absorption for different values of the top mirror reflectivity. There is no loss when* $\rho = 1$.

Achievable Phase Shift and Necessary Cavity Phase Modulation Range

Depending on the balance of the top mirror reflectivity r and the dimensionless attenuation constant α used previously in our definition $\delta = \beta + i\alpha$, there can be changes in both the range of achievable phase shifts and the necessary modulation range of the cavity phase for realizing those shifts. In particular, in examining (9) we see that there are two distinct operating regimes. The first is the condition in which the denominator (i.e. the real part of the electric field up to a constant) can take on both positive and negative values depending on the value of the cavity phase β. In this regime, regardless of the

values of α and r, the resonator will be capable of a full 0 to 2π phase modulation. The second regime occurs when for all values of β the denominator remains nonnegative, which occurs when $r + r^{-1} \leq e^{-\alpha} + e^{\alpha}$. With the restrictions $|r| < 1$ and $e^{-\alpha} \leq 1$, this condition simplifies to $r \geq e^{-\alpha}$. In this regime, at most the resonator can only impart phase shifts ranging from 0 to π; however, depending on the exact values of α and r, this range will be reduced. Under this condition, the phase curve takes on a different form from that in Fig. 1 of the main text, having a different asymmetric line shape which goes to zero at $\beta = \pi$ (Fig. S2).

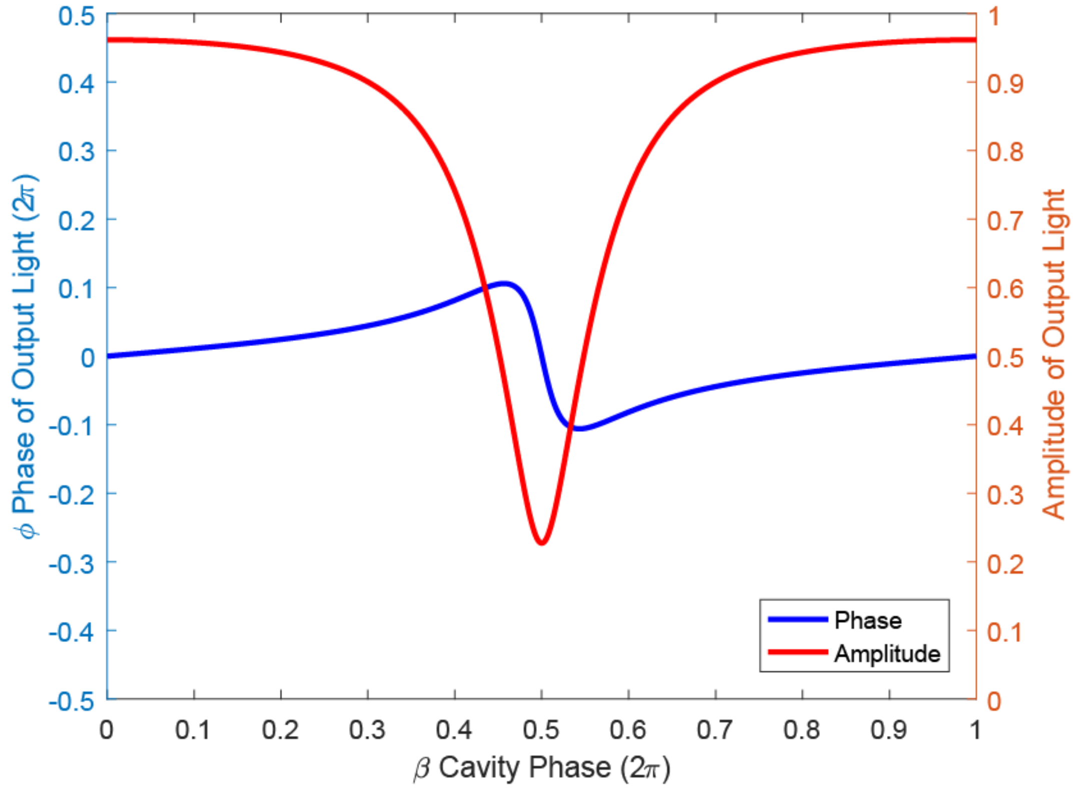

Figure S2: Phase and amplitude of the reflection coefficient for an asymmetric Fabry-Perot cavity with loss and top mirror reflectivity such that it operates in the regime $r \geq e^{-\alpha}$. Here $r = 0.8$ and $\alpha = 0.357$.

This line shape exhibits local extrema situated about $\beta = \pi$ which determine the phase modulation range. To calculate this range, we first determine the position of the local maximum by taking the derivative of the argument of the arctangent in (9) and setting it to zero.

$$\frac{\partial}{\partial \beta}\left[\frac{(1-r^2)e^{-\alpha}\sin\beta}{r(1+e^{-2\alpha})+(r^2+1)e^{-\alpha}\cos\beta}\right] = 0, (16)$$

Solving (16) for β gives:

$$\beta_{max} = \cos^{-1}\left[\frac{-(r+r^{-1})}{e^{-\alpha}+e^{\alpha}}\right], (17)$$

Due to the antisymmetric nature of the phase with respect to the $\beta = \pi$ axis, we can express the total phase modulation range $\Delta\phi$ in terms of the phase evaluated at β_{max}:

$$\Delta\phi = 2\phi(\beta_{max}), (18)$$

More generally, we can express the phase modulation range for both regimes as a piecewise function as below:

$$\Delta\phi = \begin{cases} 2\pi, & r < e^{-\alpha} \\ 2\phi(\beta_{max}), & r \geq e^{-\alpha} \end{cases}, (19)$$

Fig. S3 shows the phase modulation range for different values of r as a function of the fractional amplitude loss $1 - e^{-\alpha}$. The abrupt transitions between the two regimes are followed by rapid decays in the phase shift range.

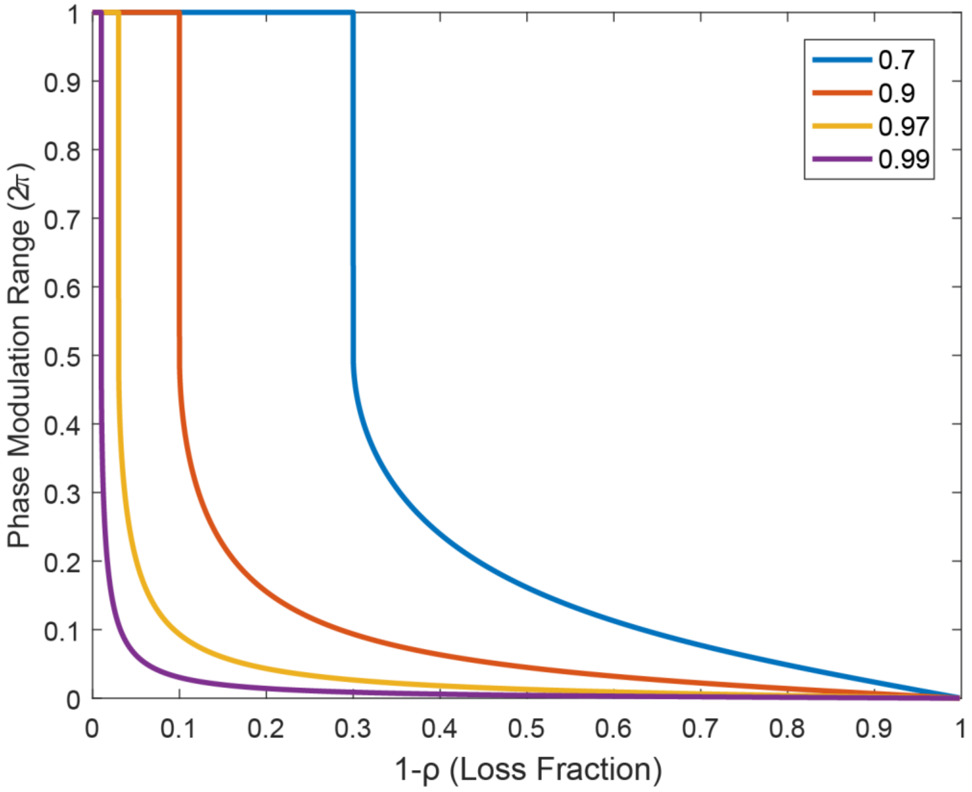

Figure S3: Phase modulation range as a function of the fractional amplitude loss for different values of the top mirror reflectivity. There is no loss when $\rho = 1$.

In addition to characterizing the achievable phase shift, it is important to quantify the necessary cavity phase modulation range. To reduce the necessary change in refractive index, this range should be minimized. From Fig. 1 of the main text we saw that by increasing the top mirror reflectivity we could reduce the necessary cavity phase range. We can quantify this range by calculating the range over which 80% of a 2π shift is achieved, as a full 2π shift would always require a 2π cavity phase range. Fig. S4 shows the required cavity phase range as a function of the fractional amplitude loss for $r = 0.7$ and we see that the required range decreases as the loss increases, but the overall change is minimal and in this particular case only corresponds to a 11.5 % change. While we

could minimize this required cavity modulation range by introducing lossy materials, in terms of realizing a practical device, the modest benefit provided by this reduction would likely be offset by the drop in amplitude efficiency due to material absorption.

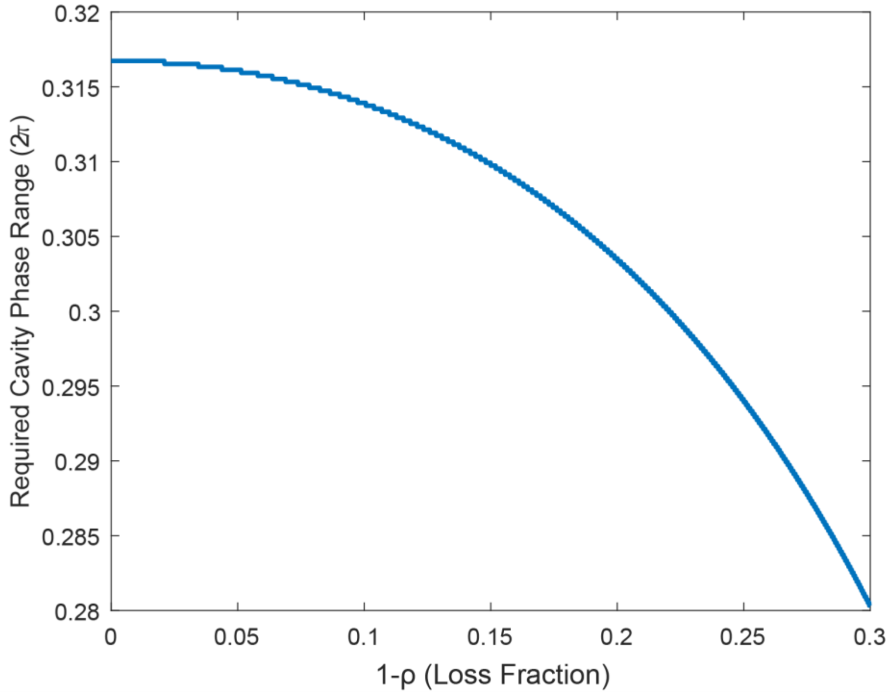

Figure S4: Necessary cavity phase modulation range as a function of the fractional amplitude loss for $r = 0.7$*. Here, the loss fraction is restricted to the range such that we are in the phase regime where 0 to* 2π *shifts are achievable, as outside this regime the notion of the required cavity phase range is ill-defined as the maximum possible shift is* π*. There is no loss when* $\rho = 1$.

Focusing Efficiency

To characterize the efficiency of our tunable metasurface aspherical lenses, we calculated the focusing efficiency as a function of on-axis focal length (Fig. S5). In particular, we took the ratio of the total power in the focal plane within a circle of radius three times the focal spot's FWHM to the total power incident on the metasurface[1]. We report a trend of

increasing efficiency with higher focal lengths, and found focusing efficiencies as high as 41% at 280 μm focal length.

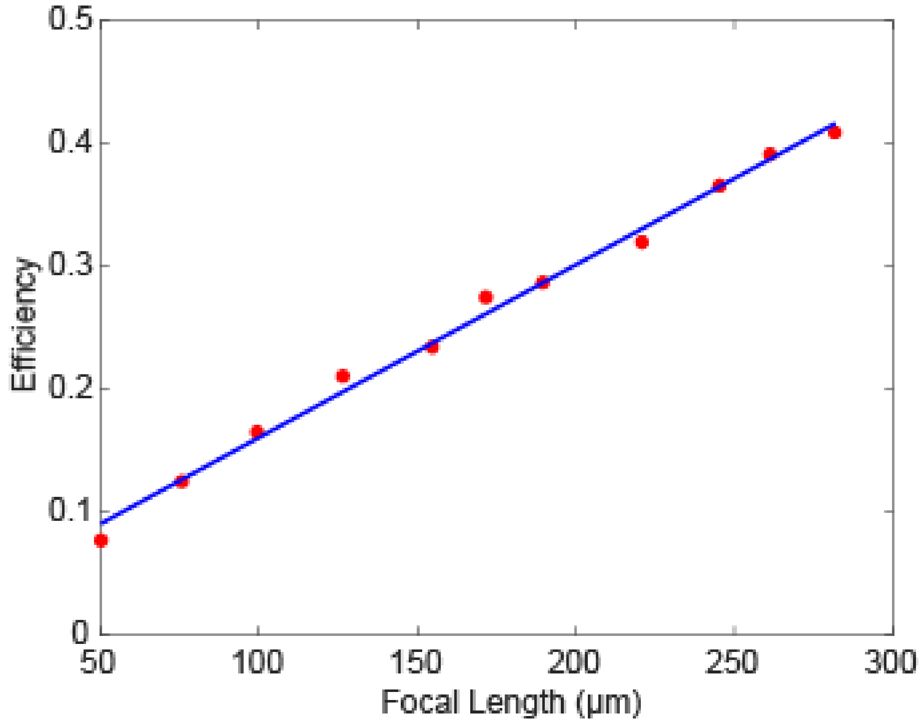

Figure S5: Focusing efficiency as a function of focal length for the tunable aspherical metasurface lens. The blue line is an eye guide.

Design of Rectangular Scattering Elements

The asymmetric structure of our nanoposts can be generalized to other scattering element geometries for which electrical routing for index tuning would be simpler. A 1-D grating of rectangular lines with a unit cell as in Fig. S6a is possible and could be used in focal line scanning applications. Fig. S6b shows the phase characteristics for such an example device structure with lines of width 620 nm, period 800 nm, and height 516 nm, achieving nearly 2π phase modulation with a change in refractive index of 0.0949 (< 3%). The incident light is polarized orthogonal to the length of the lines, which in RCWA

are modeled as infinitely long rectangles. With this structure, an 80 μm x 80 μm tunable cylindrical lens was designed, demonstrating focal scanning (Fig. S6c).

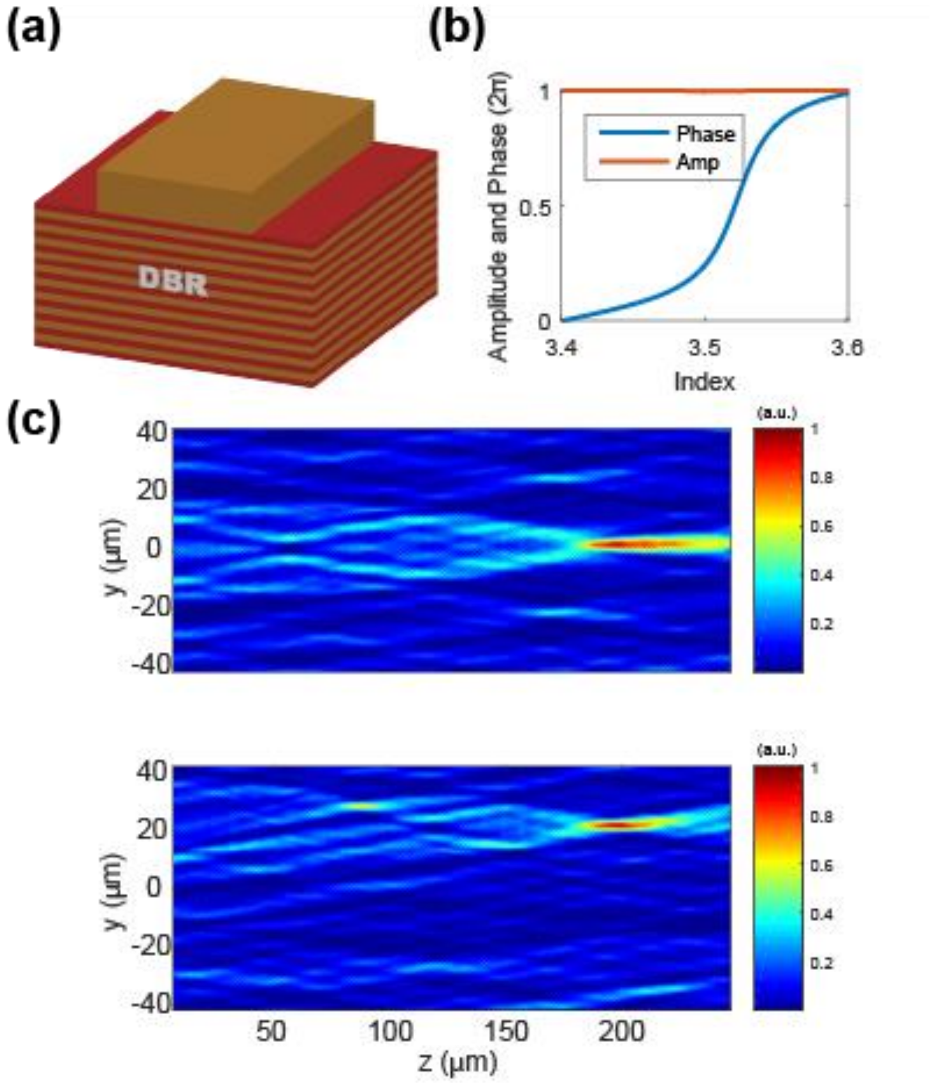

Figure S6: Scattering rectangle design: (a) Schematic of the scattering element unit cell (b) RCWA-calculated reflection coefficients as a function of refractive index (c) Intensity profiles for a tunable cylindrical lens with 200 μm focal length on-axis (top) and 20 μm off-axis (bottom)

Index Modulation by Thermal Tuning

One route to tune the refractive index of individual scatterers is by exploiting the thermo-optic effect by heating either electrically or optically. Based on the thermal coefficient of refractive index for silicon at room temperature ($\frac{dn}{dt} = 1.86 \times 10^{-4} K^{-1}$), we calculate a temperature change of ~317 K for our nanoposts:

$$\Delta T = \frac{\Delta n}{dn/dt} \approx 317\ K, \text{(20)}$$

Index Modulation by Free Carrier Injection

To calculate the change in carrier density (ΔN) necessary for nearly 2π phase modulation of our nanoposts ($\Delta n = 0.059$), we use the Drude Expansion Model[2]:

$$\Delta n = -\frac{e^2 \lambda^2}{8\pi^2 c^2 \epsilon_0 n}\left(\frac{1}{m_e^*} + \frac{1}{m_h^*}\right)\Delta N, \text{(21)}$$

where e is the electron charge, λ is the operating wavelength, c is the speed of light, ϵ_0 is the vacuum permittivity, n is the nominal refractive index, and m_e^* and m_h^* give the electron and hole conductivity effective masses respectively. Substituting appropriate parameter values, we get a carrier density change of $\Delta N = 2.93 \times 10^{19} cm^{-3}$. Under such high carrier densities, Auger recombination will dominate relative to the radiative and Shockley-Read-Hall pathways, allowing us to calculate the recombination lifetime as:

$$\tau = \frac{1}{CN^2}, \text{(22)}$$

where C is the Auger recombination coefficient. With the determined recombination lifetime, we can find the generation rate of electron-hole pairs in the steady state using:

$$G = \frac{\Delta N}{\tau}, \text{(23)}$$

If we assume the incident laser used for exciting free carriers has a wavelength of 500 nm, the absorption coefficient corresponds to an average penetration depth of nearly $1\ \mu m$, which is greater than the height of our nanoposts, allowing us to approximate a depth-uniform generation rate[3]:

$$G = N_0 \alpha, (24)$$

where N_0 is the incident photon flux and α is the absorption coefficient at 500 nm. Combining (23) and (24) and then multiplying the flux by the photon energy, we calculate a required incident laser intensity of $1.26\ MW/cm^2$ if thermo-optic effects are neglected.

We could also achieve carrier injection electrically by forward biasing a nanopost configured as a p-n or p-i-n junction diode (Fig. S7). With a p-n junction configuration, the highly doped p and n regions would enable very high changes in carrier density near the junction, which would fall off rapidly as you move away from the center. Instead, with a p-i-n junction configuration, via Sentaurus simulation we calculate that with our 504 nm thick posts, with 50 nm thick p and n regions with dopant concentrations on the order of $3 \times 10^{19} cm^{-3}$, we can achieve the necessary carrier modulation ΔN nearly uniformly across the entire intrinsic region with only 5 V applied bias. We emphasize that reverse biased p-n junctions (as opposed to our forward biasing here) generally used in silicon photonic modulators usually produce very small changes in carrier concentration over a small volume, which is not ideal for tunable metasurface applications.

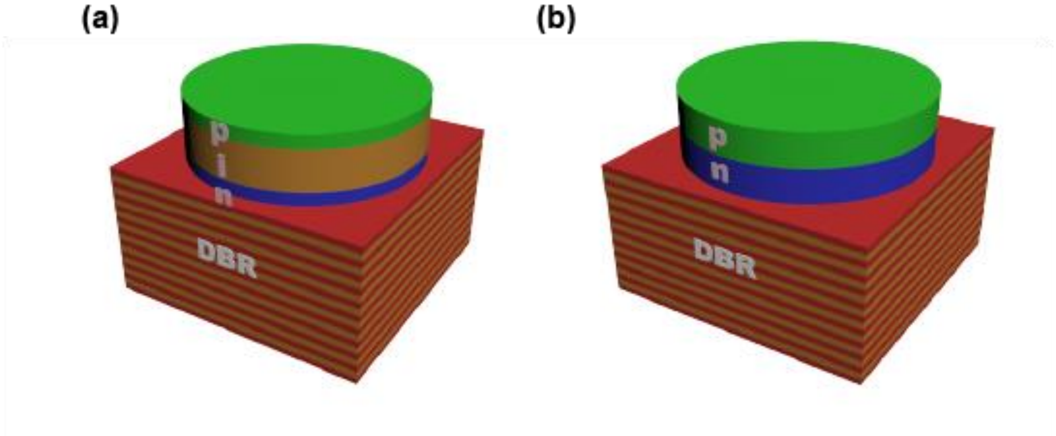

Figure S7: Experimental realization using junction diode-based scatterers. Schematic of realizable unit cells based on p-i-n (a) and p-n (b) junction diodes atop a DBR.

Phase Modulation with Multi-Element Unit Cells

As discussed in the main text, in going to subwavelength resolution with a single scattering nanopost per unit cell, we must carefully handle the resultant coupling between adjacent pixels. Depending on the level of variance in phase as a function of the lattice constant, the designed scatterers may be more well-suited for implementing particular classes of phase profiles. Our heuristically determined moderately coupled nanoposts in Fig. 2d of the main text lend themselves well to implementing lenses, axicons, and vortex beam generators, profiles with relatively low phase gradients; however, for the rapidly varying profiles necessary for generating holograms, such as that in Fig. S8a, the resultant hologram in the far-field (Fig. S8b) exhibits a missing upper corner, bright spots, and gaps indicative of errors in phase modulation. If instead we reduce the spatial resolution and utilize pixels of double the area which consist of a 2x2 set of identical nanoposts (Fig. S8d), then we can ensure all phase gradients are reduced by a factor of 2. With this reduced spatial resolution, the generated hologram in the far-field (Fig. S8c) is cleaner. While there are still bright spots, the generated hologram matches the ideal result

(Fig. S8a) more closely, an indication that with our designed nanoposts the span of achievable profiles is related to the coupling between elements and if higher phase gradient profiles are necessary, then either an improved scatterer design with minimized coupling or reduced spatial resolution is required. These holograms were designed via the Gerchberg-Saxton algorithm[4], simulated by finite-difference time-domain, and propagated to the far-field by the Fraunhofer diffraction integral. The resolution of the simulated images is limited by the available computational resources which prevent simulation of larger and higher resolution holograms.

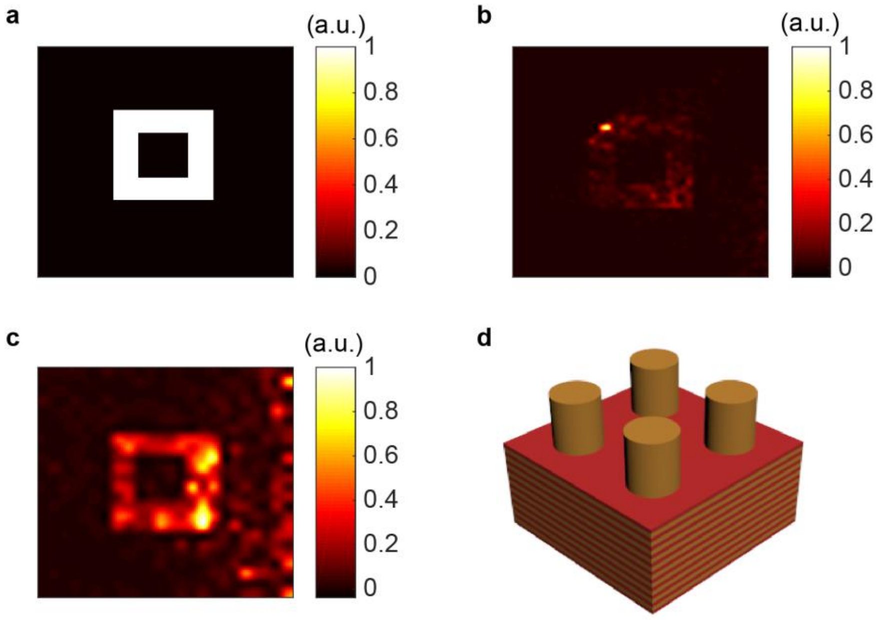

Figure S8: Hologram generation with different scattering pixel designs. (a) The ideal hologram generated in the far-field if there is perfect phase modulation. (b) The far-field holograms produced using a single resonator (b) and 2x2 resonator geometry (c) per pixel. (d) Schematic representation of the 2x2 resonator arrangement for a single pixel.

Simulation by the Angular Spectrum Method

As simulating large regions using the finite-difference time-domain (FDTD) method is memory and time-intensive, we only use FDTD to simulate the fields propagated to a few microns off our devices and then use a wave optics propagator to simulate the fields at much further distances. In particular, as our propagation distances are much smaller or on the order of our aperture size, the Fresnel number F of our system satisfies $F \gg 1$, making both the Fresnel and Fraunhofer diffraction integrals invalid. Instead, we employ the angular spectrum method[5], treating the spatial Fourier components in a given plane as a superposition of plane waves with different propagation directions. If we denote the E-field profile in the plane $z = z_0$ near the metasurface as $E_{meta} = E(x, y, z_0)$, then we can write the field in an arbitrary plane as:

$$E(x, y, z) = E_{meta} * H(k_z, z - z_0), \quad (25)$$

where $*$ denotes convolution and H gives the free-space propagator defined as the inverse Fourier transform of the reciprocal space propagator, $\widehat{H}$:

$$\widehat{H} = e^{\pm i k_z z}, \quad (26)$$

The longitudinal wavevector is a function of the wavenumber and transverse wavevectors, defined as:

$$k_z = \sqrt{k^2 - k_x^2 - k_y^2}, \quad (27)$$

References

1 Arbabi, A., Horie, Y., Ball, A. J., Bagheri, M. & Faraon, A. Subwavelength-thick Lenses with High Numerical Apertures and Large Efficiency Based on High Contrast Transmitarrays. *arXiv:1410.8261* (2014).

2 Henry, C., Logan, R. & Bertness, K. Spectral dependence of the change in refractive index due to carrier injection in GaAs lasers. *Journal of Applied Physics* **52**, 4457-4461 (1981).

3 Bhattacharya, P. *Semiconductor optoelectronic devices*. (Prentice-Hall, Inc., 1994).

4 Gerchberg, R. W. A practical algorithm for the determination of phase from image and diffraction plane pictures. *Optik* **35**, 237 (1972).
5 Goodman, J. W. *Introduction to Fourier optics*. (Roberts and Company Publishers, 2005).
